# A comprehensive approach to understand somatic symptoms and their impact on emotional and psychosocial functioning in children

**DOI:** 10.1371/journal.pone.0171867

**Published:** 2017-02-08

**Authors:** Rita Cerutti, Valentina Spensieri, Carmela Valastro, Fabio Presaghi, Roberto Canitano, Vincenzo Guidetti

**Affiliations:** 1 Department of Dynamic and Clinic Psychology, Sapienza University, Rome, Italy; 2 Department of Psychology of Developmental and Social Processes, Sapienza University, Rome, Italy; 3 Division of Child and Adolescent Neuropsychiatry, University Hospital of Siena, Siena, Italy; 4 Department of Paediatrics and Child and Adolescent Neuropsychiatry, Sapienza University, Rome, Italy; University of L'Aquila, ITALY

## Abstract

**Introduction:**

Somatic symptoms are frequently reported by children with significant impairment in functioning. Despite studies on adult populations that suggest somatic symptoms often co-occur with difficulties in identifying and describing feelings, little research has been done in childhood. This study aimed to investigate the prevalence and frequency of somatic symptoms as well as to investigate the functional impairment in children with high number of self reported somatic symptoms versus those with fewer somatic symptoms. Additionally the parental perception of their children's somatic symptoms and functioning was explored. Finally, we explored the direct and indirect effects of difficulties in identifying feelings in predicting somatic symptoms and functional disability among school-aged children.

**Methods:**

356 Italian school-aged children and their mothers participated in this study. Children (mean age = 11.43; SD = 2.41) completed the Children’s Somatization Inventory (CSI-24) to assess somatic symptoms, the Functional Disability Inventory (FDI) to assess physical and psychosocial functioning and the Alexithymia Questionnaire for Children (AQC) to evaluate alexithymic features. Mothers completed the parental forms of the CSI and the FDI.

**Results:**

Among children, 66.3% did not declare somatic symptoms and 33.7% reported one or more somatic symptoms in the last two weeks. A significant positive correlation emerged between children’s and mothers’ CSI total scores. Both children’s and mothers’ FDI total scores were significantly correlated with CSI scores. A significant correlation was observed between somatic symptoms and alexithymic features. Furthermore, the data showed that somatic symptoms mediated the relationship between difficulties in identifying feelings and functional impairment. Finally, it was showed that alexithymia facet of difficulty in identifying feelings contributed in large part to the prediction of the somatic symptomatology (b = 0.978, p < 0.001; R^2^ = 0.164, F(5, 350) = 10.32, p < 0.001).

**Conclusions:**

Findings from this study provide evidence that a higher frequency of somatic symptoms is associated with functional disabilities and alexithymic facets in school-aged children.

## Introduction

For a long time, children presenting physical symptoms without a clear medical cause, sometimes defined as medically unexplained symptoms or functional somatic symptoms, have been commonly recognized as relevant and problematic aspects of paediatric practice among clinical and non-clinical settings [1]. Both definitions appeared relatively neutral, properly descriptive, non-pejorative, and generally accepted by various medical specialities [2,3], but, in the recent years, they have been debated in light of the proposed criteria for the fifth version of the Diagnostic and Statistical Manual of Mental Disorders (DSM-5) and included in the new category of “Somatic Symptom and Other Related Disorders” [4]. These disorders are characterized by somatic symptoms (SS) causing significant distress or dysfunction and they include psychosocial factors that may be considered as symptom initiating, aggravating and perpetuating factors [4,5].

According to the DSM-5, individuals may have a combination of physical symptoms for which an organic cause can be found and symptoms for which there is no underlying medical explanation. SS must be significantly distressing or disruptive to daily life and must be accompanied by excessive thoughts, feelings, or behaviours [4]. These new criteria seem to be more in line with findings from previous studies on developmental age. However, several general limitations of a categorical approach should be considered when the focus is on a paediatric population, since SS are subjective experiences and children may have difficulty in describing the presence, concern and levels of severity of the disease. More emphasis should be laid on the relationship between child and environment, as well as the child's adaptive functioning. The effective communication between the main caregivers of the child and the child him/herself is necessary for successful management of somatic symptoms [3].

In childhood and adolescence, physical symptoms accompanied by emotional distress represent a complex and multi-determined phenomenon to deal with in clinical practice and they can lead to considerable impairment in a child’s life, influencing development, school attendance and achievement as well as social adjustment [1].

SS are common in school-aged children, with approximately 25% of children experiencing chronic or recurrent pain (e.g., headache, abdominal pain, and sore muscles) and 10% reporting chronic fatigue [6–8]. For some children, these symptoms are short-lived with no negative long-term impact on daily functioning or developmental course. However, the majority of these symptoms are associated with functional disability, emotional distress, requests for medical care and school absenteeism [5,9,10] as well as fewer hobbies, impairment in daily life, leisure and sporting activities [11].

To date, the contributions devoted to identifying the risk factors that influence the trajectories of SS and the outcomes of maladaptive child behaviour have been poorly assessed. Research has demonstrated a strong correlation between SS and alexithymia, even if most studies have been conducted on adult samples [12], in which a greater difficulty in identifying feelings appeared in conjunction with SS [13,14]. Alexithymia refers to a limited ability to identify and communicate one’ s feelings, which has been frequently associated with physical health complaints [15,16]. Alexithymia is also an important risk factor for the onset of somatic symptoms in children, even though few studies have focused on non-clinical populations [8]. Nemzer [17] highlighted that the lack of cognitive capacity and adequate emotion regulation skills may lead to SS in specific situations with a negative impact on academic and psychosocial functioning.

According to a developmental perspective and with research indicating the importance of social contest, the particular care of parents may reinforce the expression of SS [18]. Previous research [19–22] has illustrated that the correspondence between children and parents was rather modest in the case of children’s SS. More precisely, parents typically indicated fewer SS than do their children in school-based children samples [22]. Conversely, parents are likely to report more SS compared with children among clinical samples [23].^.^Moreover, studies pointed out that parents who are more supportive and sensitive may facilitate their children in successfully managing distress and coping with emotionally arousing situations [24].

In light of the above considerations, our principle aims were to:

1. determine the prevalence and frequency of somatic symptoms reported by children;
2. investigate whether there is a functional impairment in children with a high number of self-reported SS compared to those who report fewer SS;
3. analyze the parental perceptions of their children’s somatic symptoms, since health beliefs and family rules are passed on to children by their parents or other significant family members,
4. explore the relationship between SS and alexithymic features;
5. verify the hypothesis that difficulties in identifying feelings (DIF) is not only predictive of multiple SS but also of functional impairment, with an indirect effect through SS on functional impairment.

Consistent with previous research, we hypothesized that school-aged children with a high number of SS present higher levels of alexithymic features than healthy youth. Furthermore, we expected that alexithymic features predicted the risk to experience multiple SS and related functional impairment.

## Materials and methods

### Participants

Three hundred and fifty-six Italian children, 176 girls (49.4%) and 180 boys (50.6%) aged 8 to 15 years (mean age = 11.43; SD = 2.41), and their mothers (n = 356) were recruited for this study (Table 1). All participants were Caucasian. Mothers were asked about the health status of their children in a specific schedule. Children undergoing pharmacological therapy (n = 10; 2.6%), or having existing diagnosed infections or other medical illnesses (n = 22; 5.6%) or undergoing psychological therapy (n = 1; 0.2%) were excluded from the final sample. The sample of participants were part of a general non-clinical population and were recruited in primary and middle public schools in central Italy and involved in the study as part of a health promotion project. A written informed consent was obtained from the parents before inclusion in this study. Collective administration of the self-report questionnaires took place during school time in the classrooms. Mothers received the questionnaires via their children, with the request to complete measures at home and return materials in a sealed envelope. Anonymity of participants was ensured. This study was approved by the Ethics Committee of the Medicine and Psychology Faculty, Sapienza University of Rome.

**Table 1:** Distribution of socio-demographic characteristics of participants involved in the study.

	n	%
**Gender**		
** boys**	180	50.6
** girls**	176	49.4
**Age**		
** 8–10 y.o**	134	37.6
** 11–14 y.o.**	183	51.4
** 15–16 y.o.**	39	11.0
**Income**		
** 0–10.000 €**	15	4.2
** 10.000–15.000 €**	39	11.0
** 15.000–31.000 €**	119	33.4
** 31.000–70.000 €**	113	31.7
** More than 70.000 €**	14	3.9
** Not reported**	56	15.7

### Measures

#### Assessment schedule of children’s health

Mothers were asked to report upon their child’s physical and mental health status by completing a specific list of medical illnesses and/or existing diagnoses, as well as report if their child was under pharmacological and/or psychological therapy.

#### Somatic symptoms

The Children’s Somatization Inventory (CSI) [25,26] was used to assess children’s perception of SS. Specifically, the short version of the CSI (CSI-24) [27] was translated into Italian using the translation–back-translation method, with the approval of the Author. This instrument explores the presence of SS but is most commonly used to assess somatization among children and adolescents [2,28]. The CSI-24 score was computed following instructions given in Appendix I by Walker and colleagues [27]. Besides to give the reader a screenshot of the distribution of SS, we further considered the score obtained from the sum of the dichotomized CSI items as described in Walker, Garber and Greene [25]. Adequate reliability and validity of the CSI has been established. In healthy paediatric samples, internal consistency (i.e., Cronbach’s alpha) of the CSI-24 was .87 [27]. In the current study, Cronbach’s coefficient was .84. The Children’s Somatization Inventory-Parent Form (CSI-P) is identical to the child form (CSI-C), except that parents complete the questions with regard to their children’s SS during the past 2 weeks using the same response format as the child version. The CSI-P internal consistency in this sample was .82.

#### Child impairment

The Functional Disability Inventory (FDI) [29] was used to assess children’s self-reported difficulty in physical and psychosocial functioning due to their physical health. The instrument was translated into Italian with the translation–back-translation method and approved by the Author. Functional difficulties are expressed in 15 items concerning perceptions of activity limitations during the past 2 weeks, including performance of daily activities at home, school, recreation, and social situations. The FDI has good internal consistency and 3-month test-retest reliability estimates exceeding 0.60 for patients with chronic abdominal pain. In the current study, we found an internal consistency of .77. The Functional Disability Inventory-Parent Form (FDI-P) has the same structure of items and response format as the FDI-child form (FDI-C). Alpha reliability coefficients on the FDI-P ranged from .94 to .90. In the present sample, the Alpha coefficient was .76.

#### Alexithymia

In order to assess alexithymic features, children completed the validated Italian version of the Alexithymia Questionnaire for Children (AQC) [30,31]. It is a simplified version of the Toronto Alexithymia Scale (TAS-20) [32], and this questionnaire consisted of 20 items, representing 3 factors: Difficulty Identifying Feelings (DIF); Difficulty Describing Feelings (DDF) and Externally-Oriented Thinking (EOT). The internal consistency for the DIF and the DDF scales was good (Cronbach’s alpha of approximately .75), while the EOT factor did not meet the criteria for internal consistency nor item homogeneity, as also confirmed in the present study (.27). In the current study, Cronbach’s alpha was .74 for DIF and .62 for DDF.

### Statistical analyses

The SPSS 19.0 (Statistical Package for Social Sciences) software package was employed in the analyses of the data. Descriptive statistical analysis were used to describe the characteristics of participants. Pearson correlations were computed to determine the associations between somatic symptoms, child impairment and alexithymic features.

Mediation analyses were conducted to examine the direct effect of the DIF on functional disability, and its indirect effect through SS as measured by the CSI-24 on functional impairment. Mediation was tested using the SPSS macro, PROCESS. In particular, a series of linear regression models were fitted, and the size and significance of the indirect effects were estimated by a bootstrap procedure.

## Results

### Descriptive statistics

Considering the CSI score obtained from the sum of dichotomized items, approximately 66.3% (n = 236) of the total youth sample did not report SS in the last two weeks. About twenty-eight percent (n = 100) reported 1 to 3 symptoms. The remaining 5.7% (n = 20) reported 4 or more (up to 12) symptoms according to the threshold for somatization in children proposed by Escobar [2]. By dividing the sample into three groups on the basis of reported symptoms (“no symptoms” vs “from 1 to 3 symptoms” vs “4 or more symptoms”), no gender differences emerged (χ^2^ = 1.52 df = 2, p = 0.47). Table 2 shows the differences between child and mother reports with respect to the frequency of SS on the CSI-24. Children reported more frequent SS than did their mothers. In fact, 92.1% (n = 328) of mothers indicated the absence of SS compared to only 66.3% of children (n = 236) who did not report any symptoms. 7.9% of mothers (n = 28) declared that their children suffered from 1 or more somatic symptoms. Finally, no significant correlations emerged between both child-reported and mother-reported SS and age (respectively rho = 0.063, p = 0.236; rho = 0.099, p = 0.062).

**Table 2:** Frequencies and percentages of the most reported CSI symptoms for Children (N = 356) and Mothers (N = 356).

CSI ^a^ Items	Children		Parents	
	n	%	n	%
**1 Headache**	26	7.3%	8	2.2%
**2 Faintness or dizziness**	4	1.1%	0	0.0%
**3 Pain in your heart or chest**	7	2.0%	0	0%
**4 Low in energy**	16	4.5%	4	1.1%
**5 Pains lower back**	18	5.1%	6	1.7%
**6 Sore Muscles**	16	4.5%	2	0.6%
**7 Trouble breath**	5	1.4%	1	0.3%
**8 Hot or cold spells**	14	3.9%	4	1.1%
**9 Numbness or tingling**	20	5.6%	1	0.3%
**10 Weakness**	10	2.8%	2	0.6%
**11 Heavy feelings arms or legs**	6	1.7%	3	0.8%
**12 Nausea or upset stomach**	19	5.3%	0	0%
**13 Constipation**	11	3.1%	5	1.4%
**14 Loose BM's or diarrhea**	4	1.1%	1	0.3%
**15 Pain stomach or abdomen**	30	8.4%	3	0.8%
**16 Heart beating fast**	16	4.5%	1	0.3%
**17 Difficulty swallowing**	3	0.8%	1	0.3%
**18 Losing voice**	9	2.5%	0	0%
**19 Blurred vision**	8	2.2%	1	0.3%
**20 Vomiting**	5	1.4%	0	0%
**21 Floated or gassy**	14	3.9%	2	0.6%
**22 Food making sick**	12	3.4%	1	0.3%
**23 Pains knees, elbows or joints**	13	3.7%	1	0.3%
**24 Pains arms or legs**	6	1.7%	1	0.3%

Note.

^a^ CSI = Children's Somatization Inventory

#### Psychological correlates

With regards to functional disability, 5.9% of the total sample fell into the moderate range (from 13 to 29) based on the Kashikar-Zuck cut-off [33], while only 1.1% of mothers indicated a similar range regarding their children’s disability. A statistically significant difference was found between child (M = 4.61; SD = 5.28) and parent-report (M = 1.32; SD = 2.59) on the FDI total score (t (356) = 11.36, p<0.001). In line with past studies [29], higher scores on the FDI were reported by children who declared more SS (r = 0.534, p < 0.01) and a similar correlation was also found when considering mothers’ reports (r = 0.459, p < 0.01). In particular, children without SS reported a lower (t(354) = 3.26, p < 0.01) mean score on the FDI (M = 3.26; SD = 4.12) while children with 1 or more symptoms had a higher FDI mean score (M = 7.27; SD = 6.24). In the same way, mothers who indicated at least 1 or more SS presented a higher (t(354) = 2.10, p = 0.04) FDI mean score (M = 4.53; SD = 4.50) than mothers who indicated no SS (M = 1.05; SD = 2.16). Moreover, comparing the AQC scores as a function of participants reporting no SS with those that indicated one or more SS, it was found that the latter participants scored significantly higher on both the DIF factor (t(354) = 5.26, p < 0.01) (M = 12.72; SD = 3.15) and the DDF factor (t(354) = 2.75, p < 0.01) (M = 9.59; SD = 2.40) with respect to the participants reporting no SS (respectively: DIF: M = 10.93; SD = 2.99; DDF: M = 8.85; SD = 2.40). Finally, no differences emerged between the two groups when considering the EOT factor mean scores (respectively: No symptom group M = 14.58; SD = 2.44; ≥1 symptom group M = 14.68; SD = 2.63). In addition, considering correlations among the CSI-C scores and the AQC factors, we found a positive and significant correlation with both DIF (r = 0.393, p < 0.01) and DDF (r = 0.230, p < 0.01) but not with EOT (r = 0.029, p = 0.584). While considering CSI-P scores, only DIF correlated positively and significantly (r = 0.194, p <0.01) while both DDF and EOT did not (respectively: r = 0.031, p = 0.554; r = -0.003, p = 0.961). Finally, gender correlated modestly with DIF (rho = 0.160, p = 0.002) and with EOT (rho = -0.147, p = 0.006) but not with DDF (rho = 0.061, p = 0.252), while the age factor reported a significant correlation with EOT (rho = -0.187, p < 0.001) but not with both DIF (rho = 0.101, p = 0.058) and DDF (rho = 0.024, p = 0.649).

#### Mediational effect of CSI

The indirect effect of DIF through SS on functional disability was tested with the PROCESS macro for SPSS. Gender, age and the other two alexithymia factors (DDF and EOT) were introduced as covariates within the model. Results showed that the direct effect of DIF on FDI scores was positive and significant (b = 0.259, p < 0.001) when the CSI total score was not considered in the equation. However, when the direct effect of the CSI score is added (b = 0.326, p < 0.001), the direct effect of DIF becomes not significant (b = 0.115, p = 0.256). Girls showed significantly lower levels of FDI (b = -1.498, p = 0.002) than boys. Also age showed a significant negative effect (b = -0.211, p = 0.043). However, both DDF (b = -0.046, p = 0.715) and EOT (b = 0.173, p = 0.083) have no significant effects in predicting FDI scores. In general, all predictors explained about 32.4% of the total variability in FDI scores (F(6, 349) = 20.53, p < 0.001). The DIF scores had a positive and significant effect in predicting the CSI score (b = 0.978, p < 0.001; R^2^ = 0.164, F(5, 350) = 10.32, p < 0.001). Finally, the total indirect effect of DIF through CSI on FDI was significant (b = 0.319, Bootstrap 95% C.I.: 0.188–0.456) (Table 3).

**Table 3:** Direct and indirect effects of DIF^a^ on functional disability.

Measure		Coeff	SE	t	P
**FDI**^b^					
	CSI-C^c^	0.3264	0.0345	9.4512	<0.001
	DIF	0.1147	0.1007	1.1385	0.2557
	Sex	-1.4976	0.4876	-3.0715	0.0023
	Age	-0.2115	0.1041	-2.0306	0.0431
	DDF^d^	-0.0457	0.1252	-.3653	0.7151
	EOT^e^	0.1726	0.0992	1.7400	0.0827
**CSI-C**					
	DIF	0.9776	0.1900	5.1456	<0.001
	Sex	1.2002	0.8960	1.3395	0.1813
	Age	0.2856	0.1866	1.5309	0.1267
	DDF	0.1082	0.2102	0.5147	0.6071
	EOT	0.1061	0.1821	0.5830	0.5603

Note.

^a^ DIF = Difficulty in Identifying Feelings

^b^ FDI = Functional Disability Inventory

^c^ CSI-C = Children's Somatization Inventory-Child Form

^d^DDF = Difficulty in Describing Feelings

^e^ EOT = Externally Oriented Thought.

## Discussion

It is well known from the literature that somatic symptoms are common in childhood and that they are closely related to mental and emotional symptoms, both in the general population and in primary care populations [34]. These symptoms are associated with a diminished quality of life and involve difficulties in psychological and social functioning [35]. Findings from the present cross-sectional study, highlight that a great portion of children (n = 120) declared at least one somatic symptom during the last two weeks, and providing evidence that a higher frequency of SS is associated with higher functional impairment. This result is comparable to those of a recent study involving a school-based sample of children [35], in which youths with more SS had greater impairment in their activities at home, school and in relationships with peers [36, 37].

The present study evaluated the degree of parent-child agreement regarding children’s SS and functional disability, emphasizing the use of a multi-informant approach. Studies regarding parent-child agreement on SS and psychosocial functioning are few and agreement differs among clinical and non-clinical populations. Previous studies have revealed how children who exhibit recurrent SS miss significantly more days of school than healthy children and frequently give up gym and school trips, confirming higher scores on the FDI-C [38–40]. Our findings support the relationship between SS and impaired functioning, showing a significant correlation between CSI-C and FDI-C as well as CSI-P and FDI-P scores.

An association between child and parent scores on both the CSI and FDI was also observed. While the correlations indicate similarities between the rank orders of scores assigned to children by themselves and their mothers, the mean differences yield information about family agreement. In accordance with previous studies [19,22] our data showed a discrepancy between children and mothers in the assessment of children's SS and functional disability, with mothers perceiving a lower number of symptoms in respect to their children’s self-reports, as well as a low parental awareness concerning their children’s distress and disability [19]. These results have a clear clinical relevance because underscore the importance of considering the level of parental awareness about SS affecting their children and the related impact on psychosocial functioning in the planning of a comprehensive intervention to improve the physical and psychological well-being of children.

Furthermore, results from the multiple regression analyses highlighted that SS made an independent contribution to the prediction of functional impairment, playing a role in mediating the relationship between DIF and functional disability. The associations between SS, psychopathology and adaptation have been widely investigated, even if little research has been done on the relationship between alexithymia and psychosocial functioning [41]. With regard to the presence of somatic or medical symptoms, alexithymia has been frequently observed in association with a variety of psychopathological conditions leading to poor coping abilities which, in turn, may lead to an impairment in psychosocial functioning [42].

Interestingly, we found that SS played a role in mediating the relationship between DIF and daily functional disability. This result is new to the paediatric literature while the direct effect of DIF on SS and on functional disability have already been highlighted. In fact, data from a recent study showed DIF was associated with health-related maladaptive behaviours as well as the use of alcohol and drugs in a sample of young healthy men [43]. DIF has also been shown to be positively correlated with maladaptive patterns of immature defences and ineffective coping styles, both in non-clinical and psychiatric samples [43–45] and seems to be a significant predictor of psychopathology, particularly somatization, in psychiatric patients [45], whereas the other two facets of alexithymia, as defined by the TAS-20, showed no significant influence on psychopathology.

The strengths of this study include the use of established and standardized assessment measures and the use of multiple informants in order to explore children’s physical and psychosocial impairments that made it possible to add new knowledge to the existing literature. However, several important limitations warrant consideration. First, our sample consisted of self-reported healthy children, so it is not clear whether the results may be generalized to other populations (e.g., with chronic physical illnesses or pain). This study is cross-sectional and, consequently, the conclusions drawn should be considered with caution. Finally, participants’ understanding of the exact definitions of the symptoms along with the unknown IQ level of children (that is known to be linked to higher alexithymia scores) may have affected their answers. In particular, it cannot be excluded that lower IQ may have affected the indirect effect of DIF on functional scores through somatic symptoms.

## Conclusion

In summary, more knowledge regarding the association between SS, functional impairment and alexithymia in children may aid in the early identification and prevention of diverse negative developmental experiences and functional limitations which adversely affect school attendance and promote the development of unhealthy social relationships. The current study provides further support for the hypothesis suggesting a link between alexithymic facets, SS and psychosocial functioning in children. Future research clarifying the relationship between alexithymia and SS among young people both in clinical and non-clinical populations is needed.
